# Cholera Infantum—Read before the Erie County Medical Society, January, 1862

**Published:** 1862-02

**Authors:** Henry Nichell


AND REPORTER.

VOL. I. FEBRUARY, 1862. NO. 7.

### ORIGINAL COMMUNICATIONS.

ART. I—*Cholera Infantum—Read before the Erie County Medical Society, January,* 1862, *by* Henry Nichell, M. D. *Published by vote of the Society.*

*Mr. President and Gentlemen:*

In response to your invitation, I have to present the subject of Cholera Infantum, and will ask your indulgence, while I read an article too lengthy for the time usually devoted to an address. The importance of the subject is my only apology. I desire to call your attention to this subject, which, although almost exhausted by the most eminent and distinguished writers of our country, is in an etiological as well as pathological point of view at least, still open for further research and inquiry. I frankly confess that I enter upon the subject I have for many reasons selected, with some distrust, as to my ability to do the justice its great importance seems to require; but having earnestly reflected upon some of the causes, and also the primary and secondary or accidental complications of that disease, which I regret to say every year extinguishes the flame of life of so great a portion of our infantile population, I shall attempt to lay before th s learned and honored Medical Socie'y of the County of Erie the results of these reflections. I am also preparing a statistical record of four hundred and seventy-five cases of Cholera Infantum, having occurred in my private practice, during the period of four successive years since 1858, which I desire respectfully to submit to the Society at some future time.

Cholera Infantum, may be defined an endemic of tenderest age, making its appearance exclusively during the hot season, in the large and densely populated cities of the United States, and being characterized by vomiting, purging, and more or less rapid emaciation. In a restricted sense of the term, cholera infantum, appears to be a well marked, severe form of gastro-intestinal catarrh, either of an acute or chronic character. The disease chiefly occurs between the age of three months and two years, but also earlier and somewhat later, and is for many tangible reasons one of the most fatal affections to which infancy is exposed. The duration of cholera infantum varies from a few hours to several days, weeks, or even months, according to the nature, and more or less continued action of the causes or conditions which produce it.

Causes.—All writers on the subject agree that a combined action of various predisposing and exciting causes are requisite in order to develope cholera infantum. The conditions necessary for the production of the disease in question, are mainly, a high atmospheric temperature, confined and impure air of large and over-crowded cities, improper diet, and a certain infantile age, representing both the period of dentition and that of the physiological development of the muciparous follicles in the intestinal tube. It is easily understood that neither of the causes just mentioned appears to be alone sufficient, for it never occurs, as it is stated, in the pure air of country places during the hot summer months, except perhaps in some mitigated form, nor does it prevail in large cities during cold weather, and so far as improper diet is concerned it will frequently be borne at any season, save the hot, with surprising impunity. I shall, however, consider more fully the morbid effects of each of the causes, so as to be enabled to test their practical value, both to the practitioner and the public in general. There can be no doubt as to the essential influence of a high atmospheric temperature, to the formation of the disease, since it is shown by the fact that it prevails always in proportion to the heat of the season. It is further proved by careful observation that the appearance of a few cold days after a period of constant hot weather more or less diminishes, while on the other hand any increase in temperature will certainly increase the number of cases. Finally, according to Dr. Hexamer, cholera infantum occurs as soon as the average temperature has reached sixty-nine degrees, and ncreases with the rise of the thermometer; it is at its height at from seventy-one to seventy-eight and disappears when the temperature falls below sixty-five.

Confined and impure air, such as is found in large and densely populated cities is another cause to be mentioned. It is due to the generation of what is commonly called animal poison or animal effluvia; the exact composition of which is not yet positively known, but that sulphuretted hydrogen is among its elements appears highly probable. In order however to appreciate the real consequences, which the noxious qualities of impure air will have upon the tender infantile organism, we must bear in mind the circumstances under which animal effluvia are generated. It is a known fact that the disease is most frequent among the infants of the poorer classes, who for the most part inhabit ill-constructed and ill-ventilated dwellings, located in overcrowded lanes and alleys, often surrounded by filth and animal decomposition of every kind. The inmates of such unhealthy localities are moreover compelled to be confined and crowded together at night in their small, damp and musty sleeping apartments where from the decomposition of their exhalations and excretions, these noxious effluvia will at once be developed. It is from such sources chiefly, where, the "malaria of crowded places" actually exist, that the disease almost always receives and manifests its more dangerous character and, manifold complications.

Improper infantile diet, in addition to the causes already mentioned should be regarded as the very torch by which action, the disease is most frequently kindled and kept alive for an indefinite period. It is, however, a strange fact, that this cause so powerful in its operation, has not yet received that full attention and appreciation in an etiological point of view, among authors and the profession at large which it so evidently deserves. I venture to assert, that at least two-thirds of the cases which came under my observation and care in an extensive private practice, for a period of thirteen years have given satisfactory evidence to me, of having been excited, or else assumed a chronic character, or even occasionally led to a fatal end, by the operation of great errors in diet. It appears to prevail among certain classes of society, whose offspring are the most liable to be attacked, and where, not only an entire ignorance, but also a fearful indifference, nay, even a stubborn opposition relative to the care, and to all dietetic and hygenic rules, proper to apply to young infants, is found to exist. It is astonishing what kinds of articles, even during the continuance of the disease the unfortunate nurslings often receive as nutriment, as for instance pieces of sour apples, pickles or sausages, any kind of berries, cider, beer, in short anything they desire. Prof. Jacobi remarks the following about improper food as a cause of cholera infantum: "The well developed cases of severe gastro-intestinal cartarrh (cholera infantum) are more frequently in the second and third half year, but according to my observation just as many occur before the first year as after. Thus neither the eye-teeth nor the 'second summer' are to be blamed as much after all. But there is some truth in the blame thrown upon the second summer. A child born in the course of the winter or spring is from fourteen to twenty months old in his second summer; he is probably weaned, and then liable to gastric and intestinal disorders, depending on being fed, over fed and badly fed, or he is kept at the breast by the too careful mother, who is fearful of some imaginary constitutional disturbance. The infant, consequently, is forced, all summer to take improper food, the nutriment of a nursling, inappropriate for the digestive organs of a boy with from twelve to sixteen teeth in his mouth."—*Am. Med. Times. page* 162, 1861.

Prof. Wood also says: "That diet has much influence upon the origin and severity of the complaint, is proved by the fact, that children fed by the bottle, or the spoon, are more frequently and dangerously affected than those nourished at the breast." According to my experience, the disease once cured will certainly return again and again when mother's substitute inappropriate diet, instead of complying with proper dietetic principles.

Finally, a certain infantile age must be added to the causes of cholera infantum—of this, I shall speak hereafter, when I come to consider it, in connection with what is called dentition, which has, up to this time, been erroneously regarded as such a causative agent. Upon the well-known fact, that a certain glandular development of the mucous membrane of the intestinal tube takes place, just about the period during which the gradual formation and protrusion of teeth occurs, in consequence of which, under certain circumstances, a series of pathological conditions is apt to follow, and on ground of a strong supposition that, *only* during this period, cholera infantum makes its appearance, is based the idea that, consequently, dentition must be one of its causes. In this sense, the latter is regarded, by various excellent writers, as the "*conditio sine qua non*" of the disease. Indeed, it seems highly convenient for writers to look on dentition as a predisposing cause of cholera infantum, without any further consideration, reflection or evidence. Dr. Williams, in his principles, page 39, uses the following language, which seems to have some relation to the present subject: "The fact is simply, that the division of causes ordinarily adopted among pathologists is conventional, and convenient, rather than natural and philosophical. What are called causes, are really circumstances that are essentially and invariably antecedent to disordered action." In order to settle this question and arrive at positive conclusions, the nature and further relation of causes should be carefully and thoroughly investigated. In relation to the point in view, it appears clear to the medical mind, that, at a certain age of infancy, two distinct physiological processes take place simultaneously—one of these consists in a certain glandular development in the intestinal canal, while the other exhibits the gradual formation and protrusion of teeth. As a very natural result of the developement of the muciparous glands or follicles, their activity and power of secretion is, found to be increased more or less. In this manner, the mucous membrane may become predisposed, sooner or later, to take on morbid action. When I consider more fully what appears to be the true pathology of cholera infantum, I shall be further prepared to show, by illustration, the very distinctive relations which these two different physiological processes may have, to the disease in question. Can the gradual development and protrusion of teeth produce any serious disease whatever? Has dentition ever been a cause in the production of croup?—Although the larynx and trachea are placed anatomically so near the seat of war, where, in very young infants, Dame Nature threatens "horribile dictu!"—the protrusion of teeth, sooner or later. Indeed, every physician would smile at the very thought of the bare possibility of it. I shall never believe dentition to be a cause of any serious disease; and it is on this occasion, and before this enlightened medical society, that I pay my highest respect to Prof. Jacobi, of New York, that high minded gentleman, who first undertook, the great and scientific work, to sweep away that very ancient and stupid story or apparition, which is generally called dentition, and its imaginary concomitants, in the shape of several hundreds of terrible diseases. I herewith refer to his very important and instructive lectures on dentition and its derangements—a treatise on the subject, which does not meet its parallel in any country. Secondly, it must be borne in mind, that cholera infantum is by no means exclusively confined to that period in which teething is apt to occur, as has erroneously been stated. For my own part, I am satisfied that I have often attended infants between four and twelve weeks of age, a period of infantile life, in which, of course, what is denominated dentition does not take place.

It should also be recollected, that very young infants are not only subjected to the prostrating effects of a high atmospheric temperature, during the hot season, in general, since they are mostly confined to the rooms, and placed either in their cradles, or at their mother's breast; they are, besides this, compelled to suffer from that concentrated heat reflected from the walls of the apartments, and finally, these little creatures are exposed to the hot and often disagreeable exhalations from the individuals who have care of them, and with whom they are in so close personal contact.

In connection with this, another important fact may be mentioned, as first stated by Dr. J. Steward, which further deserves the attention of the profession. He says: "It is the deterioration of the mother's milk, from the debilitating effects of the atmospheric influences already considered.—The mother suffers as well as the child; and it appears quickly in the secretion of the milk, manifested principally in its lessened quantity, while the process of nursing is one of fatigue and exhaustion. Even if it is difficult to prove any alteration in the chemical qualities of the milk, yet the fact is well established, that the milk will undergo changes, at times, of such a nature as to impart injury to the child. We have proved this by withdrawing the child from the breast, although in general opposed to weaning children that are sick."—Dr. J. Steward's prize essay, page 279. Indeed, it appears by no means a wonder, that so delicate creatures, who are exposed to the noxious effects of atmospheric and other influences in various ways, during the hot summer months, should be attacked by cholera infantum, especially if we take into account, that they, only, and not children of an older age, are subjected to such a series of evils. No less distinguished authority than that of Dr. J. Steward, has asserted that children, having passed the period of dentition, will, if exposed to the causes already considered, almost always be attacked by dysentery instead of cholera infantum.

But the key to the mysterious fact just alluded to, why children of an older age will suffer from dysentery instead of the disease under consideration, appears to be, that rarely ever are they to be found in just the same situations and relations as those delicate nurslings are constantly placed, especially during the hot season: wherefrom it may be safely concluded that, since they are rarely exposed to the same concentrated noxious influences, so the resulting disease will be quite different, dysentery appearing instead of cholera infantum. How different, indeed, are the conditions and relations of older children, in comparison with those of an infant of more tender age; instead of being kept in constant confinement, or held closely in the arms of perspiring mothers, the bold little boy, and the lively girl, may enjoy themselves heartily in the open air, playing around in every possible manner for the most part of the day. When under such circumstances, they are not attacked by cholera infantum—they are, however, exposed to the common causes of dysentery.

From various important reasons it may be inferred, that dentition, as such, cannot further be claimed as a predisposing cause of cholera infantum, inasmuch as the process of teething has nothing to do with it whatever.—From the fact that this process is liable to become disturbed under the influence of those noxious agents which tend to develope cholera infantum, it is easily understood that it cannot act as one of such agents itself, still less can the period during which this process is going on, be regarded as a causative agent, for the reason, first, that cholera infantum does not exclusively occur during a period usually denominated that of dentition. Secondly, this period represents in nowise this process of dentition alone, but, also, the period during which another physiological process takes place of much greater apparent influence in the production of the disease, than the process of the formation and protrusion of teeth can ever be supposed to exert.—The term, " a certain infantile age," which represents and includes both the period of dentition, as well as that of the physiological developement of the muciparous follicles of the intestinal tube, should, for these reasons, be substituted and considered as a predisposing cause of the disease, instead of what is called the period of teething. Prof. Jacobi, in reference to causes of cholera infantum, remarks: " In order to prove that there is no necessity in resorting to dentition as a kind of scapegoat to explain the diarrhœa of infantile age, we need but enumerate a number of such causes on which diarrhœa is universally acknowledged to depend. The more numerous these causes are, the less necessity there is for dentition to shoulder the blame in every case. The less so, as there are but two principal connecting links between the protrusion of a tooth and the intestinal catarrh. These principal links are, either nervous irritation or the undeniable sympathy between distant parts of the same tissue. But we have seen the scarcity of cases of local stomatitis, or rather gingivitis, during the protrusion of a tooth; and certainly, we cannot expect an inflammation, which is not, to give rise to a catharrhal process that is. Injurious injesta are the prominent causes of diarrhœa in children. Purgative medicines; maternal milk overloaded with, or deficient in fat, salts, or caseine, or affected by mental emotions; artificial feeding with amylacea, either too soon or too late, or decomposed; superabundance of sugar in the food; retention and putrifaction of particles of food in the mouth; retention of a sugar solution from a sucking bag; the mere change of nurses in weaning; those and other causes will suffice to give rise to very obstinate diarrhœa indeed. Especially the time of weaning is a dangerous one in this respect.

Another very important cause of diarrhœa is high temperature. You know that dentition will take place any year, or month, or season, but you are also aware of the fact that the occurrence of diarrhœa is very much influenced by season and temperature. We know for instance, that the cases of intestinal catarrh, with or without catarrh of the stomach, especially the severe ones known by the name of cholera infantum, will frequently appear in New York about the middle or end of June, will reach their highest number in July and August, will diminish in September, and disappear in October. [*Am. Med. Times, page* 161, *Sept.* 1861.

*Anatomical Characters.*—In the acute form, a hyperaemic state of the mucous membrane, enlarged and perhaps inflamed follicles, will frequently be met with. Sometimes not even the slightest trace of inflammatory action, but an unusual paleness of the mucus surface will be found, especially if death had occurred early, as is stated by Dr. Condie of Philadelphia. In the more chronic cases there may always be found more or less vascularity and also thickening of the mucus coat, particularly of that portion, lining the large intestines, occasionally partial softening may be observed. The follicles are generally found to be enlarged, often inflamed and occasionally ulcerated; the liver may appear either quite healthy, or also in a diseased state as more or less congested, enlarged or fatty, as has variously been stated. The substance of the brain may sometimes be found congested, or there may be serious effusion in the ventricles; in some instances thickening and opacity of the arachnoid membrane will be found.

*Pathology.*—Very different opinions seem at the present time to exist among writers, so far as one or the other tissue or organ is primarily affected. Quite recently opposition was made upon a point, already thought "a settled and absolute dogma," namely, that cholera infantum is invariably associated with more or less derangement of the liver. Most of our eminent writers, as Drs. J. Steward, Eberle, Wood, A. J. Fuller and others, are as it appears firm believers in the theory, and have indeed manifested great pains to demonstrate, that through the agency of the combined influences of a heated atmosphere along with the peculiar malaria of crowded cities, during the period of dentition the hepatic organ chiefly must suffer, and for this reason doubtless be involved in the disease, so as to present either a congested or torpid state, or a certain enlargement or fatty degeneration. Dr. Steward in his prize essay on cholera infantum, page 301, says: "The disease therefore exists mainly in the mucous follicles, and in the liver." Dr. A. J. Fuller regards the disease of the follicles as a secondary affection, caused by the congested state of the liver, by which a free passage of the blood is prevented. *(Report on Treatment of Cholera Infantum in the transactions of the Am. Med. Association, vol. ix.)* Prof. Wood is of the opinion that the disease consists essentially in an irritation or inflammation of the alimentary mucous membrane, directed especially to the mucous follicles and associated with a congested or torpid state of the liver, probably depending on the same cause. *(Practice of Medicine, page* 706, *vol.* 1.*)* The long adopted and sanctioned doctrine however, based upon a certain number of cases in which the liver was found to be in a diseased or altered condition, has at length become a matter of doubt among members of the profession. Thus the post-mortem examinations made in the nursery and child's hospital in the City of New York of late years, have led to quite opposite results relative to the question whether the liver is invariably and principally involved in each and every case of the complaint. The report on treatment of cholera infantum says: "In some cases, this organ has been found fatty, but with this exception it has uniformly been healthy." *(Am. Med. Times, page* 223, *Sept.* 1860.*)* That the liver is not severely affected in every instance is proved by the fact that cholera infantum, more or less dangerous in its character, may attack the same children for two or three successive years, as I had opportunity to observe. From this it might be supposed that the liver would during the continuance of the third attack, at least have undergone such very important changes, that recovery was entirely impossible, which however took place in some cases even under the most unpromising circumstances.

The absence of biliary secretion in the alvine evacuations has by writers in general, been regarded as a positive evidence that the liver must be more or less seriously involved in the disease. But according to my own and others experience, in the majority of instances, it seems to indicate merely a functional disorder. Even in the more protracted cases of the complaint in which this symptom is firmly believed, by some excellent authors, to depend principally upon certain degrees of enlargement of the liver, the hepatic secretion will, I venture to affirm, often reappear, sooner or later, in the alvine discharges, since it is a fact that patients have recovered that were regarded quite hopeless. Structural or anatomical derangement of the liver, is most probably the result of a long-continued disorder of this organ, gradually produced, or it may also be developed in consequence of a previous existing predisposition or tendency to organic liver disease, favored by the attack of cholera infantum. The important question, why the liver appears to be involved more or less seriously in some cases, while in others this organ is found in a quite healthy condition, must be left for further investigation.

I shall now discuss more fully what appears to my mind the true pathology of cholera infantum. Either of the physiolgical processes developed during a certain age of infancy, already considered, may, through the powerful agency of various noxious influences become disturbed in one way or the other. The process of the development of the muciparous follicles, will gradually become altered into a pathological condition, and serves as a large basis of operation in cholera infantum; while the process of teething, as such, may perhaps be prolonged, and locally become more or less difficult, in consequence of exhaustion and prostration. The process of the follicular development, after having been interrupted in its physiological nature, seems to become thus morbidly modified, and not only an undue enlargement, but also, as further consequence, even inflammation, and occasionally ulceration will occur during the course of the disease. These important local effects, have been explained, by some distinguished writers, as being solely effected through the agency of the liver—the latter, on post-mortem examination, however, has, in many instances, been found quite healthy. I am inclined, therefore, to consider the mucous membrane, and the follicular arrangement of the alimentary tube, as primarily affected in cholera infantum, contrary to the opinion of many others.

The affection of the brain and its membranes is a secondary or accidental complication, in most cases at least, although morbid conditions of this organ occasionally occur during the early stages even of the disease. They are, however, the more well-marked in confirmed cholera infantum.

[TO BE CONCLUDED IN NEXT NUMBER.]

---

—Professor Peaslee recently performed the operation of tapping on a young lady at Pittston, Pa., and removed *one hundred and forty-nine pounds and three ounces* (149 lbs., 3 oz.,) of dropsical fluid. The abdominal circumference of the patient before the operation was *six feet and two inches.* This is the same patient from whom Dr. Peaslee removed *one hundred and thirty-five pounds* of fluid, (135 lbs.) on the 29th of April last. The circumference then was five feet seven inches.

